# Designing Survey-Based Mobile Interfaces for Rural Patients With Cancer Using Apple’s ResearchKit and CareKit: Usability Study

**DOI:** 10.2196/57801

**Published:** 2024-09-26

**Authors:** Alyssa Donawa, Christian Powell, Rong Wang, Ming-Yuan Chih, Reema Patel, Ralph Zinner, Eliah Aronoff-Spencer, Corey E Baker

**Affiliations:** 1 Ming Hsieh Department of Electrical and Computer Engineering University of Southern California Los Angeles, CA United States; 2 Department of Computer Science University of Kentucky Lexington, KY United States; 3 Department of Human and Organizational Development Vanderbilt University Nashville, TN United States; 4 Department of Health and Clinical Sciences University of Kentucky Lexington, KY United States; 5 Division of Medical Oncology Markey Cancer Center University of Kentucky Lexington, KY United States; 6 Division of Infectious Diseases & Global Public Health School of Medicine University of California San Diego San Diego, CA United States

**Keywords:** usability, usability testing, digital literacy, ehealth literacy, digital divide, mobile health, mHealth, patients with cancer, rural health, distress, apps, ehealth adoption, HealthKit, CareKit

## Abstract

**Background:**

Despite the increased accessibility and availability of technology in recent years, equality and access to health-related technology remain limited to some demographics. In particular, patients who are older or from rural communities represent a large segment of people who are currently underusing mobile health (mHealth) solutions. System usability continues to hinder mHealth adoption among users with nontraditional digital literacy.

**Objective:**

This study aims to investigate if state-of-the-art mobile app interfaces from open-source libraries provide sufficient usability for rural patients with cancer, with minimal design changes and forgoing the co-design process.

**Methods:**

We developed Assuage (Network Reconnaissance Lab) as a research platform for any mHealth study. We conducted a pilot study using Assuage to assess the usability of 4 mobile user interfaces (UIs) based on open-source libraries from Apple’s ResearchKit and CareKit. These UIs varied in complexity for reporting distress symptoms. Patients with cancer were recruited at the Markey Cancer Center, and all research procedures were conducted in person. Participants completed the distress assessment using a randomly selected UI in Assuage with little to no assistance. Data were collected on participant age, location, mobile app use, and familiarity with mHealth apps. Participants rated usability with the System Usability Scale (SUS), and usability issues were documented and compared. A one-way ANOVA was used to compare the effect of the UIs on the SUS scores.

**Results:**

We recruited 30 current or postsurgery patients with cancer for this pilot study. Most participants were aged >50 years (24/30, 80%), from rural areas (25/30, 83%), had up to a high school education (19/30, 63%), and were unfamiliar with mHealth apps (21/30, 70%). General mobile app use was split, with 43% (14/30) of the patients not regularly using mobile apps. The mean SUS score across the UIs was 75.8 (SD 22.2), with UI 3 and UI 4 achieving an SUS score ≥80, meeting the industry standard for good usability of 80. Critical usability issues were related to data input and navigation with touch devices, such as scale-format questions, vertical scrolling, and traversing multiple screens.

**Conclusions:**

The findings from this study show that most patients with cancer (20/30, 67%) who participated in this study rated the different interfaces of Assuage as above-average usability (SUS score >68). This suggests that Apple’s ResearchKit and CareKit libraries can provide usable UIs for older and rural users with minimal interface alterations. When resources are limited, the design stage can be simplified by omitting the co-design process while preserving suitable usability for users with nontraditional technical proficiency. Usability comparable to industry standards can be achieved by considering heuristics for interface and electronic survey design, specifically how to segment and navigate surveys, present important interface elements, and signal gestural interactions.

## Introduction

### Background

Mobile health (mHealth) technologies have been around for over a decade, yet the percentage of adult patients actively using these mHealth technologies is lower than desired [1,2]. The demographics of adults not using mHealth solutions are consistent with patients from rural populations, racial and ethnic minority groups, and older individuals, which overlaps with persons categorized as medically underserved [3]. According to the Health Resources and Services Administration, medically underserved populations have been designated as having too few primary care providers, a high infant mortality rate, prevalent poverty, or a high older adult population [4,5]. Specifically, rural communities, such as those of the Southeastern United States or Appalachia, commonly have higher rates of chronic disease, reduced access to providers, and fewer medical resources than their urban counterparts [6-10]. The ubiquity of mobile devices makes mHealth particularly attractive for reaching populations that are disadvantaged [11-14]. A promising use of mHealth is remote patient monitoring, which can include objective data, such as biometrics via sensor devices, or subjective data, such as quality-of-life surveys via patient-reported outcomes, resulting in a better understanding of a patient’s overall health and symptom tracking between visits [15,16].

As of 2023, a total of 90% of people in the United States own a smartphone. In addition, it was reported that while 27% of people who lived in rural areas did not have broadband at home, 87% owned a smartphone [17,18]. In particular, adopting innovations in rural communities is essential because the disparities between advantaged communities and those that are disadvantaged continue to grow for digital literacy [16,19,20], also known as the digital divide [20-22]. Factors in the divide between advantaged groups and those that are disadvantaged are health literacy, knowledge of technology, and comfort of use [20,23,24]. Designers should ensure that system user interfaces (UIs) are universally acceptable, particularly concerning users with limited technical proficiency [20]. Ensuring the usability of a system is essential for accurate data collection and reducing attrition rates [25-27].

Simply digitizing a paper-based survey may present complexities that render the digitized counterpart unusable and discourage the required frequency and accuracy necessary to improve adherence [28-30]. For example, patients might accidentally submit their responses prematurely or fail to submit them at all. In addition, usability plays a crucial role in the adoption of innovative technologies, as explored through the technology acceptance model [31-33] and research on mHealth adoption [27,34-36]. A participant’s age has been shown to substantially affect the ease of navigation and learnability, especially as cognition and motor control decline [37]. However, proper interface design can minimize user error and allow a smooth user experience [38]. To address the aforementioned concerns, researchers and developers can co-design the UI to ensure digitization is tailored to the respective demographic [39,40].

### Distress Screening

**Figure 1:**
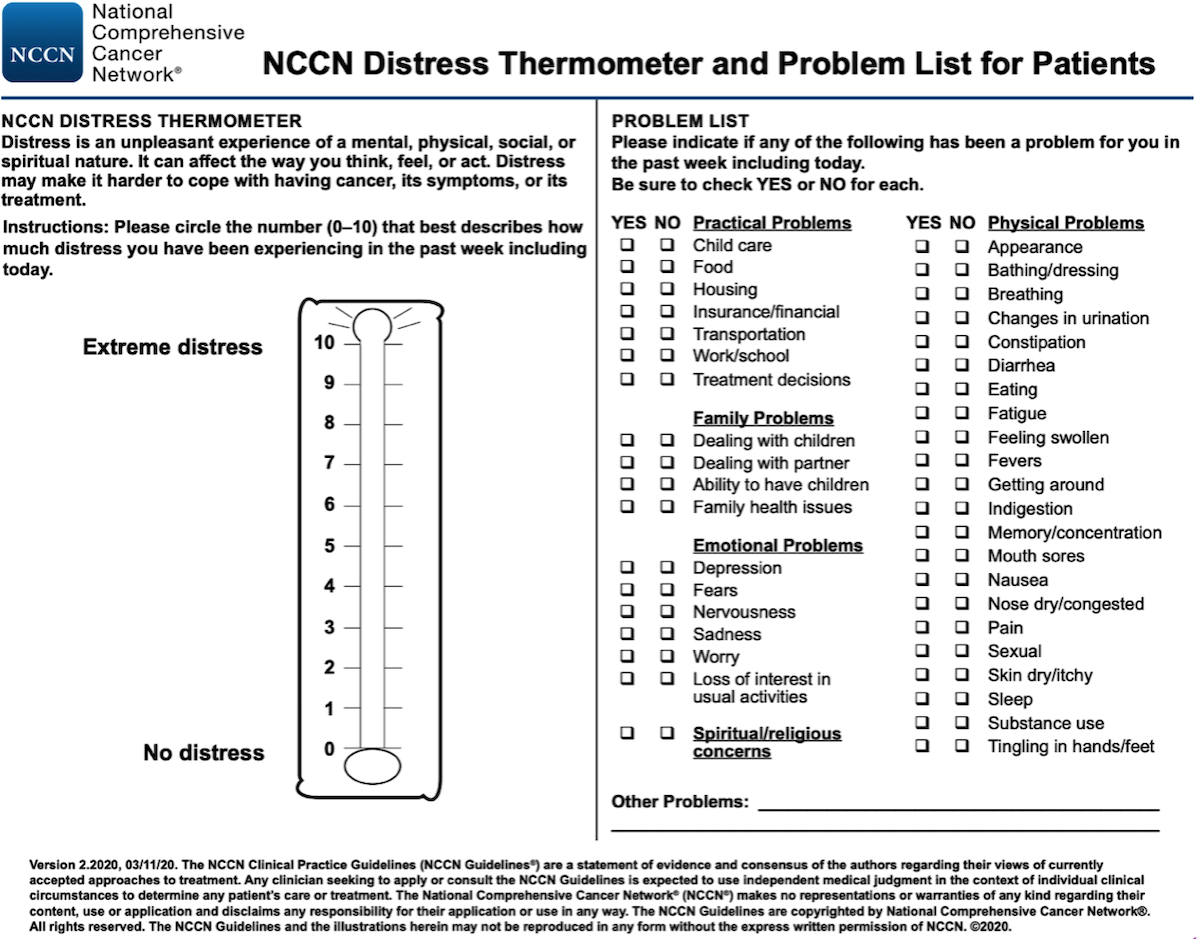
National Comprehensive Cancer Network’s (NCCN) Distress Thermometer and Problem List. The version shown here was the version used for this study. The newest version can be found in the NCCN guidelines [41].

According to the National Cancer Institute, distress is an “emotional, social, spiritual, or physical pain or suffering that may cause a person to feel sad, afraid, depressed, anxious, or lonely” [41]. Distress is prevalent in patients with cancer regardless of disease stage or modality [8,42-45], and untreated distress has been shown to lead to greater pain, reduced physical function, increased medical costs, and longer stays in the hospital [8,42,46]. The National Comprehensive Cancer Network (NCCN) created the Distress Thermometer and Problem List, hereafter referred to as the NCCN assessment, for use as a screening tool for recognizing distress in patients with cancer (Figure 1) [47,48], and has since been shown to indicate distress accurately [42,49]. The NCCN assessment was designed to improve patient care and increase patient quality of life. Furthermore, studies have shown that distress screening can improve health outcomes, including reduced morbidity and mortality [8,42].

Unfortunately, due to factors such as staff burnout or emotional fatigue, signs of distress in patients may go unnoticed [42,43]. In addition, there can also be variations across different cancer centers regarding when patients should be screened [42]. This raises the need for a more effective and efficient process related to distress screening [43]. The implementation of the NCCN assessment as a mobile app poses many advantages, such as real-time identification of distress factors and triage to the proper provider, generating insightful data around common issues during the cancer experience, and providing insight into potential resource allocation [14,15,50].

Conversely, there are barriers to the implementation of new tools in health care. For example, modifying any clinical practice can be challenging, and providers hesitate to make drastic changes without sufficient evidence of substantial benefit and patient-driven motivation [51-53]. In addition, digital implementations of distress screening that are considered complex or not user-friendly by target users can lead to reduced effectiveness. Effective distress screening requires patient adherence and accurate information input to enable providers to devise proper interventions and follow-ups [14]. Despite the challenges, technology poses a great solution to address the needs of patient distress monitoring when resources and access to care are limited [54,55]. In particular, the prevalence and ubiquity of mobile devices present opportunities for patients in remote and rural areas to use mHealth apps to enhance their care. By reducing the time between distress screenings, providers and researchers can improve their understanding of a patient’s overall distress and track symptoms between visits.

### Open-Source Frameworks

In 2014, Apple launched HealthKit, a central repository for health and fitness data that is automatically available on all iOS devices, and subsequently launched an open-sourced ResearchKit in 2015. Three major modules make up ResearchKit: informed consent, surveys, and active tasks [56,57]. Institutions such as Duke and Stanford have launched research-based mobile apps using ResearchKit [58]. Mobile apps developed using ResearchKit have already begun to be integrated into standard hospital software systems such as Epic Systems [59]. The ResearchKit framework has been used in various mHealth apps, including those focused on asthma, autism, Parkinson disease, type 2 diabetes, cancer, cardiovascular issues, mental health, pregnancy, postpartum conditions, hepatitis, and epilepsy [56].

In 2016, Apple released and open sourced a complementary framework to HealthKit and ResearchKit, called CareKit (Apple Inc), which supports personalized health care with customized care plans, adherence tracking, and visualization of trends in user data [60]. CareKit consists of 3 independent modules: CareKitUI, CareKitStore, and CareKitFHIR. CareKitUI provides a set of health, fitness, and medical views that can be customized to create mobile apps. CareKitStore provides local storage of patient data on personal devices using CoreData, which is Apple’s implementation of an SQLite database. Data generated using the CareKitStore framework are securely stored and encrypted on the device [61]. CareKitFHIR enables seamless conversion between CareKitStore objects and Health Level 7 Fast Healthcare Interoperability Resources [62] resources to integrate with Fast Healthcare Interoperability Resources–based electronic health records and applications. Combining HealthKit, CareKit, and ResearchKit allows for the development of mHealth apps with many desired features for remote patient monitoring and self-management of health by users [26,63] with reduced effort from developers [60]. Together, these iOS and iPadOS frameworks enable the collection and sharing of user-generated health data and streamline the process of building survey-based mobile apps for research [64].

### Survey Design Heuristics

The following heuristics from previous research can be followed to provide an optimal user experience for respondents in digital surveys. Surveys should be aesthetically pleasing, easy to navigate [28,30], and have an explicit visual flow [65]. Although some researchers [66] have found that scrolling layouts can sometimes have faster completion times, designers should still be strategic in deciding between paging versus scrolling along with the grouping and sequencing of questions. Furthermore, when considering answer choices, potential options should include some variation of “do not know” [67,68]. In addition, surveys should be succinct [30,65,69] and maintain a standardized format, as variations in format can lead to decreased usability [28]. Surveys should always be easy to understand, with clear directions for answering questions [28,65,69]. Moreover, survey language should mimic verbal dialogue whenever possible [28]. Additional features to consider implementing are showing participants their progress toward completion, a *thank you* page, and an overview of results at the end [28].

A set of usability heuristics often used as a baseline for designing systems is the 10 principles for interaction design postulated by Nielsen [70], that consist of the following guidelines: (1) visibility of system status; (2) match between system and real world; (3) user control and freedom; (4) consistency and standards; (5) error prevention; (6) recognition rather than recall; (7) flexibility and efficiency of use; (8) aesthetic and minimalist design; (9) recognize, diagnose, and recover from errors; and (10) help and documentation. Finally, incorporating the 10 general principles for interaction design by Nielsen [70,71] will make UIs more accessible, user-friendly, and intuitive.

### This Study

Ensuring the interface usability of an mHealth system is essential to its effectiveness, which often requires patient adherence and accurate information input to enable providers to devise proper interventions and follow-ups and prevent attrition [27,72]. Previous research suggests that co-designing for users with limited digital literacy, such as older or rural users, may be required to build suitable usable interfaces, but it often requires considerable time and resources [73-75]. Designers often co-design the UI to address the concerns and ensure digitization is tailored to the respective demographic [40]. This pilot study assesses the usability of multiple UI implementations of the NCCN assessment (Figure 1), particularly for understudied populations such as Appalachian and rural patients with cancer who are underserved and vulnerable [76,77]. The different UIs were designed without co-design to assess whether usable UIs could be achieved for this demographic when resources for the design stage are limited.

## Methods

### Ethical Considerations

The University of Kentucky’s Institutional Review Board approved all research activities (approval number: 64149). Informed consent information was provided to participants with a cover letter, and participants confirmed their consent to participate in the study after an in-app onboarding and consent process. Participants could withdraw from the study at any time. Data were collected anonymously and stored on encrypted servers. Participants were not compensated for taking part in this study.

### Recruitment

Patients with cancer were recruited in person from the University of Kentucky’s Markey Cancer Center to participate in this study between July and August 2021. Two medical oncologists at the cancer center permitted us to interact with willing patients at their clinics. The physicians asked if patients would be willing to speak to a researcher about the study during their visits. If patients agreed, we went to the respective waiting room; informed the patients about the purpose of the study; gauged interest; and, if applicable, proceeded with the study tasks. If patients were not interested in the study, we thanked them for their time, and they were not entered into the pilot study. We recruited 30 patients to participate in this study. Participants did not need to have a certain level of digital literacy, as we were interested in participants who were not very familiar with mobile devices and apps to assess whether Assuage would be usable for people with limited digital literacy. Participants were not offered payment to participate in this study.

### Procedure

This pilot study used between-groups A/B testing to compare the usability of 4 different UI designs for completing a distress survey in a mobile app. A/B testing, or split testing, is a randomized experiment where users are shown ≥2 versions of a system, website, or app to determine which version performs better based on specific metrics [78]. A/B testing protocols are commonly used in industry, and different system versions are randomly assigned to users for comparative analysis [78,79]. All research procedures were conducted in person at the Markey Cancer Center. Assuage was preinstalled onto an iPad for participants to use. After we went over informed consent information with the patient, the procedure went as follows: we asked patients the following demographic questions: age range, sex, ethnicity and race, education, residence, familiarity with the paper form of the NCCN assessment, mobile app use frequency, and mobile apps for health and medical use frequency.

We then introduced Assuage to the patient, which reiterated consent via an in-app onboarding process and study information and reverified that the patient was still interested in participating. Assuage was programmed to randomly select one of the UIs to display to users following the in-app onboarding. This was done by assigning a number from 0 to 3 to the different UIs and randomly selecting an integer in that range. Therefore, we did not have direct control over which UI group participants were assigned. Participants were presented with the randomly selected UI and instructed to follow the app prompts to complete the distress assessment. If a participant went through the NCCN assessment with a companion, the participant did all the physical interaction with the interface. It was appropriate for 7% (2/30) of the patients to enlist the help of their accompanying caregiver, as this mimics assistance needed naturally in the clinical or at-home setting.

While participants were completing the NCCN assessment in the app, we observed and collected notes on any usability issues, software bugs, and other noteworthy information. If a participant got stuck or confused using the app, we gently nudged them on how to proceed and documented the usability issue. After participants finished using Assuage to complete the assessment, they completed a usability assessment. Afterward, participants were asked to provide additional comments regarding the study and their use of Assuage. We also inquired about each participant’s specific set of mobile devices. No identifiable participant information was collected through the Assuage app. No video or audio recordings took place. Notes about the participants’ actions, usability issues, and responses were also collected, and usability issues were organized into related themes.

### Outcomes Measured

**Figure 2:**
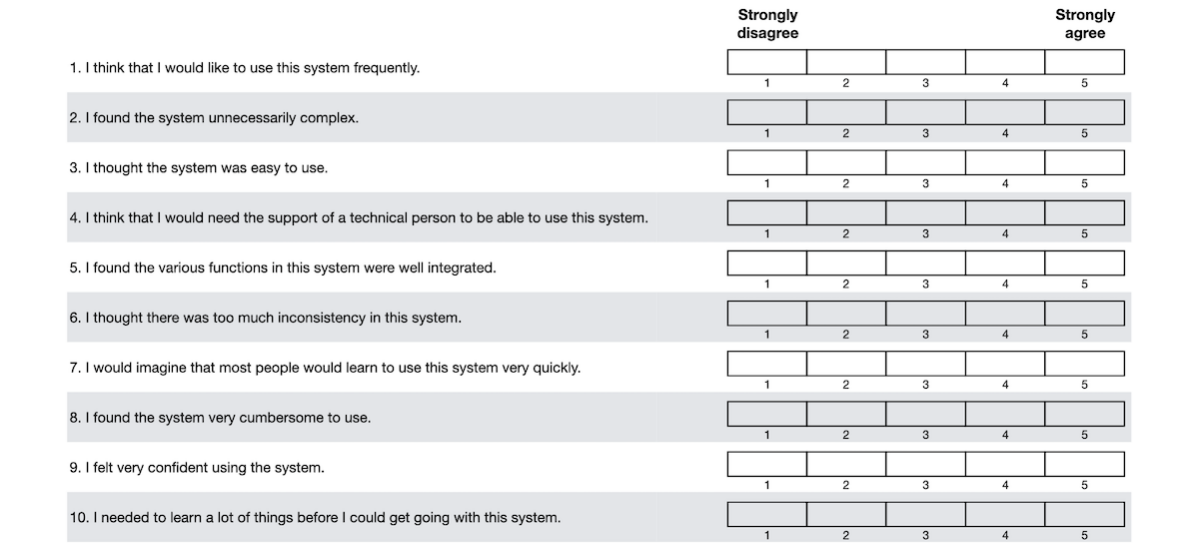
System Usability Scale [80].

This study measured perceived usability by participants after completing the distress assessment with Assuage. Scores from the System Usability Scale (SUS) [80] were compared among the UI design variations within Assuage. The SUS is a validated tool with a reputation for providing swift and reliable results [81,82]. The SUS consists of 10 statements, or items, with a 5-point Likert scale ranging from 1 (strongly disagree) to 5 (strongly agree). A negative response is considered a score <3 for positively worded statements and >3 for negatively worded statements. While the SUS is not a diagnostic tool, it can effectively determine whether the tested system would be generally usable even when used to evaluate small sample sizes with as few as 5 users [80-86]. The SUS has been used in industry and academic research and is sufficient for pilot studies of mHealth apps [25,27,40,83,87-89]. Individual SUS items are provided in Figure 2.

The SUS scores from participants (N=30) were grouped by the respective UIs tested by the participants, and the results were analyzed using the *SciPy* Python (version 3; Python Software Foundation) package in iPython Notebooks [90]. A one-way ANOVA was performed to compare the effect of the different UIs on usability, represented by the SUS score. Lewis and Sauro [81] assessed data from 241 usability studies to create a curved grading scale where an SUS score of 68 is a “C” grade and considered acceptable usability. However, industry targets an SUS score of 80 to represent an above-average user experience [81]. We used a content analysis approach to analyze qualitative data, such as observed usability issues and participant comments. Content analysis is a method used to systematically classify data, usually written, into segments with codes (labels) to make inferences about the content and underlying themes [91]. Data were coded using Taguette, which is a free and open-source qualitative tool [92].

### System Design and Development

Assuage is a Health Insurance Portability and Accountability Act (HIPAA)–compliant mobile iOS, iPadOS, and watchOS platform developed using Apple’s HealthKit [93], CareKit [60], and ResearchKit [56]. Assuage is a research test bed for assessing and improving patient care through health-related studies. Remote patient monitoring can be accomplished through Assuage by adding various quality-of-life surveys, such as the NCCN assessment in Figure 1. Additional frameworks such as ParseCareKit [94] synchronize ResearchKit and CareKit data with a HIPAA-compliant server [95]. Assuage offers multiple UIs for patient input of subjective information such as their distress symptoms. The decision to provide multiple UIs is based on the knowledge that some demographics, such as rural patients with cancer, have not heavily adopted mHealth but are also not completely removed from modern everyday technologies, such as mobile devices or smartphones [17,18,96]. Conversely, the number of rural-dwelling adults who own a smartphone continues to rise [18], creating avenues for mHealth to have a larger impact on this population. Therefore, we wanted to gauge if standard UI elements common in mobile interfaces provide acceptable usability for an mHealth use case, such as symptom reporting, without expending extra resources on co-design.

**Figure 3:**
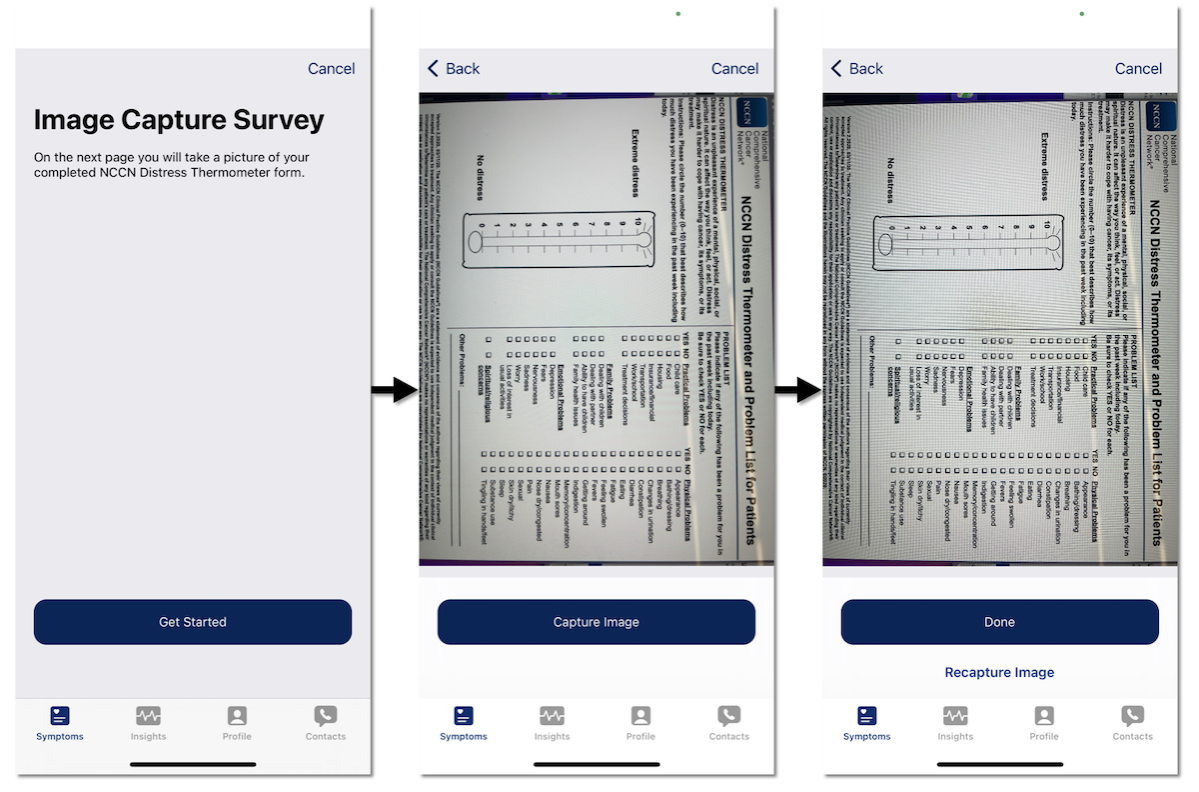
User interface 1.

**Figure 4:**
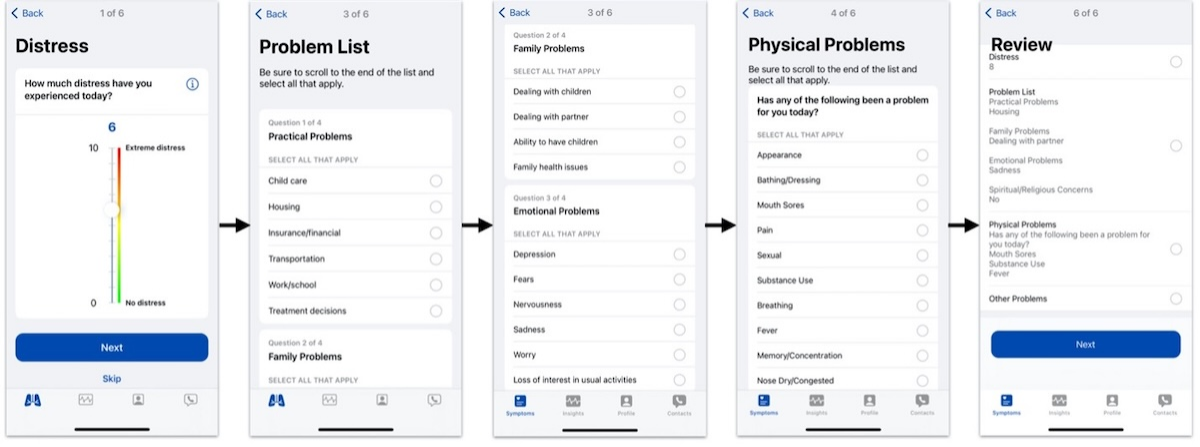
User interface 2.

**Figure 5:**
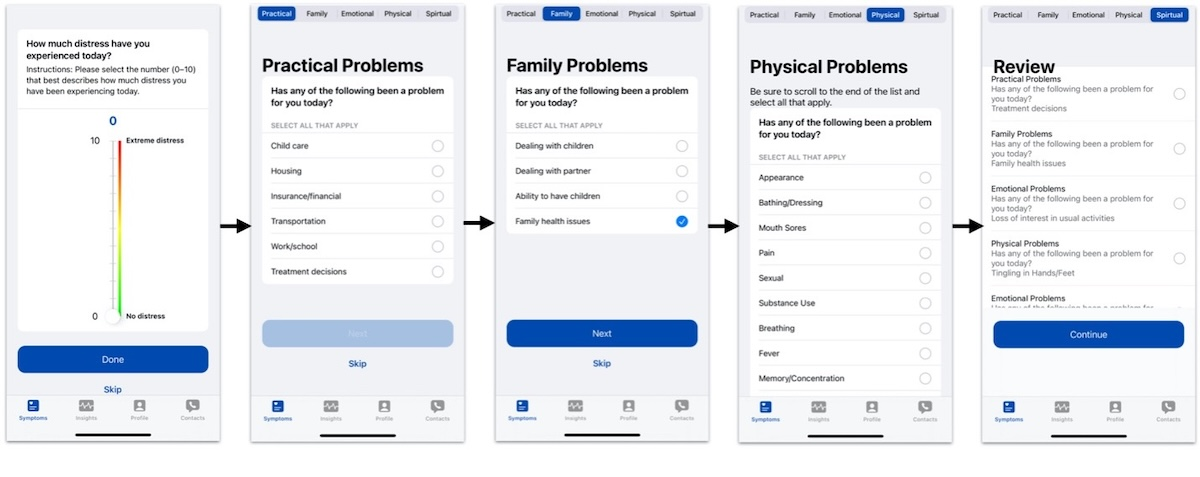
User interface 3.

**Figure 6:**
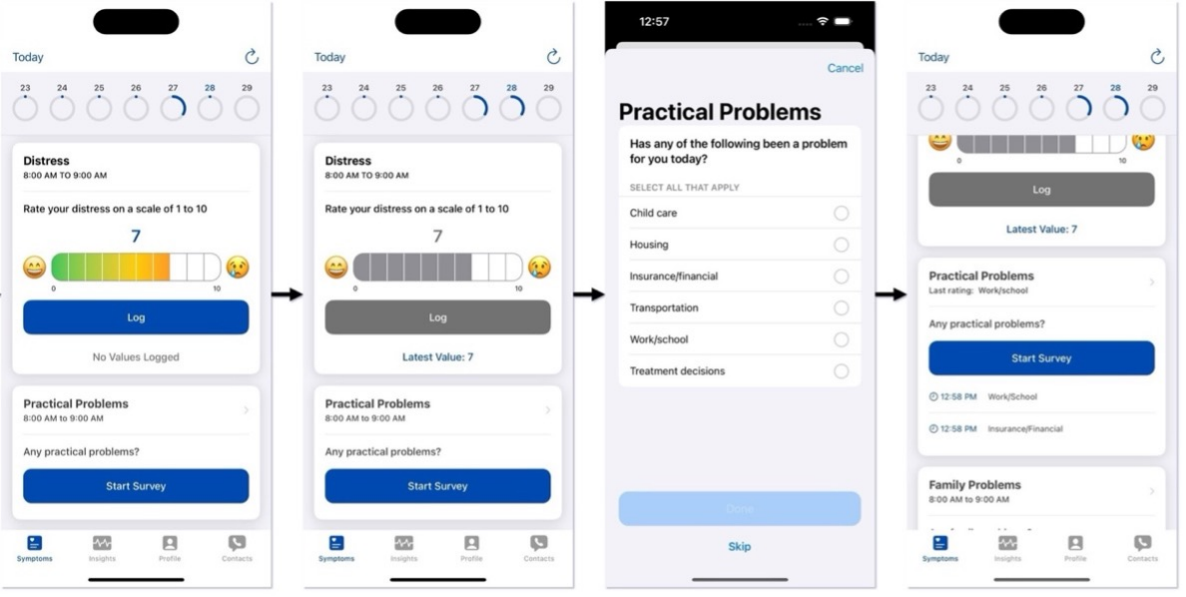
User interface 4.

Four UIs were implemented in Assuage for the pilot. All the UIs were designed with Apple’s ResearchKit and 1 with CareKit, which leverages Apple’s Human Interface Guidelines [97]. ResearchKit and CareKit provide out-of-the-box UI views and elements for developers to build health and medical mobile apps, which have been used in various research studies [34,64,98-101]. Screenshots of the different UIs are shown (Figures 3-6). In particular, the UIs differ in how the NCCN assessment components are displayed and navigated. When gauging a patient’s distress using the NCCN assessment, the reported value of the distress thermometer component typically correlates to the immediate actions taken by the care team regarding the patient. With this in mind, the entirely digitized interfaces (UIs 2-4) present the distress thermometer first, but patients can still choose to skip any question in all UI versions. Descriptions of the different UIs are presented in Textbox 1.

**Descriptions of the different user interfaces (UIs).**

**Descriptions**

1. UI 1 (Figure 3): enables patients to sequentially step through 3 screens to capture a picture of the paper-based National Comprehensive Cancer Network (NCCN) assessment. UI 1 depends solely on ResearchKit’s (Apple Inc) standard survey design with no alterations. Navigation is limited to the next and back buttons. Patients familiar with the NCCN assessment survey may benefit from UI 1 because it requires completing the paper-based survey as normal. Conversely, if a patient is unfamiliar with mobile devices or has ailments that prevent them from holding the camera steady, UI 1 could be less usable.
2. UI 2 (Figure 4): patients navigate the NCCN survey components sequentially. UI 2 depends solely on ResearchKit’s standard survey design, with no alterations. Navigation is limited to the next and back buttons. Patients unfamiliar with the NCCN survey may benefit from UI 2 because all the NCCN survey questions must be viewed before completing the survey. Conversely, the sequential requirement of UI 2s design does not allow the user to quickly navigate different survey sections compared to the paper NCCN assessment or UI 3 and UI 4. This may require more time to be spent on the survey and could burden patients already familiar with the NCCN survey question set, who prefer to skip sections that do not apply to their current distress. When a patient reaches the end of UI 2, they can review their answers before submission and are allowed to change previously entered questions.
3. UI 3 (Figure 5): patients can navigate the NCCN survey sequentially and nonsequentially with a horizontal navigation segment, allowing patients to skip around to different sections. UI 3 is designed by retrofitting ResearchKit’s survey design with a horizontal navigation segment that enables patients to skip around to the different sections of the NCCN survey, providing improved navigation. In addition, UI 3 requires minimal vertical scrolling by the patient compared to UI 2. Similar to the paper-based NCCN assessment, UI 3 allows patients to quickly see all relevant distress categories. However, unlike the paper-based survey, patients are not overwhelmed by having to step through all the questions and are only presented with questions associated with the respective section of interest. Patients familiar with the NCCN assessment may benefit from UI 3’s design as it allows quicker survey completion times because they can navigate to sections and questions of interest. On the contrary, if a patient is unfamiliar with the NCCN assessment or a patient who is familiar forgets a relevant question to their distress belonging to a particular segment label, skipping around may cause questions to be missed, reducing the ability of the care team to provide the best care. When patients reach the end of UI 3, they can review answers before submission and change previously entered responses.
4. UI 4 (Figure 6): the UI implements a modern and modularized view of the NCCN assessment and is highly dependent on vertical scrolling. Patients can select cards corresponding to surveys, allowing for the most fluid navigation between sections. The navigation and card layout in UI 4 leverages both ResearchKit and CareKit (Apple Inc) and takes advantage of the latest iOS design principles. The distress thermometer in UI 4 keeps the thermometer aesthetic of the paper-based NCCN assessment but deviates by being placed horizontally instead of vertically. In addition to the temperature and number values that UIs 1 to 3 have on the distress thermometer, UI 4 also has emojis representing extreme distress points. UI 4 allows patients to scroll through survey sections vertically, while answers provided on previous days can be viewed by swiping the screen horizontally. Individual survey cards display the answers entered for the respective survey section. An adherence circle is also shown at the top of UI 4 to represent survey completion. Limitations to UI 4 are similar to those of UI 3, concerning patients unfamiliar with the NCCN assessment who may miss recording relevant answers. In addition, if a patient is not comfortable with the latest UI design principles of iOS, patients could be deterred from UI 4.

To reiterate, the most significant change in design among the different UIs is the navigation style and how a user will traverse through the survey in the app. Regarding mHealth tracking apps for users with chronic illness, the design should be simple, self-explanatory, visually appealing, and intuitive to navigate [30].

**Figure 7:**
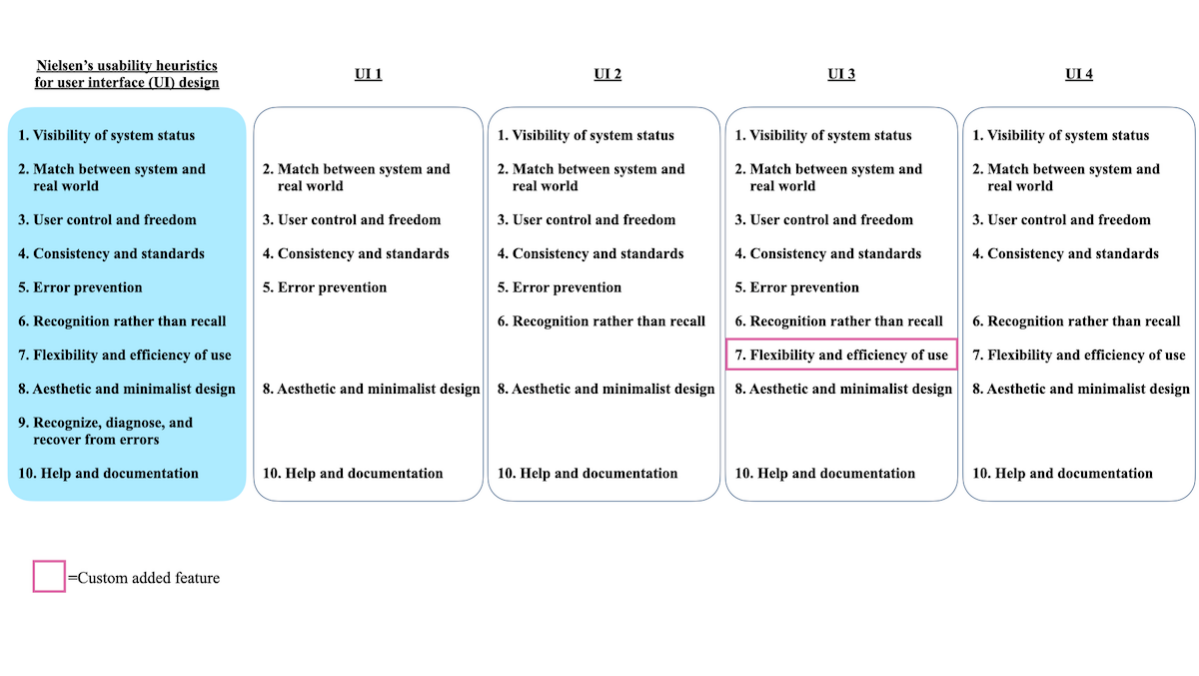
The 10 usability heuristics by Nielsen and the different heuristics covered in Assuage’s 4 user interfaces (UIs).

Figure 7 highlights which of the usability principles by Nielsen were present in the different UI variations. Note that 3 out of the 4 UIs used out-of-the-box interface elements, while we customized the navigation elements of UI 3 to include a flexible survey navigation method. This customization aligns with the guideline by Nielsen for flexibility and efficiency of use (#7) and is supported by prior research on electronic survey navigation design [28,30]. Further description of how the different UIs in Assuage satisfied the usability heuristics can be found in Table S1 in Multimedia Appendix 1.

## Results

### Overview

This section presents the findings of this pilot study regarding the 4 UIs. Descriptive statistics are reported in Table 1. A total of 30 usability surveys were completed across Assuage’s 4 UIs. This study was not designed or powered to detect the differences among the UIs; therefore, the comparative results reported should be considered preliminary evidence [40].

**Table 1:** Participant demographics^a^.

Variable		UI^b^ 1 (n=6), n (%)	UI 2 (n=8), n (%)	UI 3 (n=7), n (%)	UI 4 (n=9), n (%)	Total (N=30), n (%)
**Sex**						
	Female	3 (50)	4 (50)	3 (43)	6 (67)	16 (53)
	Male	3 (50)	4 (50)	4 (57)	3 (33)	14 (47)
**Age (y)**						
	>50	5 (83)	6 (75)	7 (100)	9 (100)	24 (80)
	≤50	1 (17)	2 (25)	—^c^	3 (33)	6 (20)
**Race and ethnicity**						
	Non-Hispanic White	5 (83)	6 (75)	7 (100)	9 (100)	27 (90)
	Non-Hispanic Black	1 (17)	2 (25)	—	—	3 (10)
**Education**						
	Did not complete high school	—	—	3 (43)	1 (11)	4 (13)
	High school	3 (50)	5 (63)	2 (29)	5 (56)	15 (50)
	Some college	1 (17)	2 (25)	1 (14)	2 (22)	6 (20)
	College degree	2 (33)	1 (13)	1 (14)	1 (11)	5 (17)
**Mobile apps**						
	Never or rarely	3 (50)	3 (38)	4 (57)	3 (33)	13 (43)
	Sometimes or more	3 (50)	5 (63)	3 (43)	6 (67)	17 (57)
**Health apps**						
	Familiar	2 (33)	3 (38)	1 (14)	3 (33)	9 (30)
	Unfamiliar	4 (67)	5 (63)	6 (86)	6 (67)	21 (70)
**Residence**						
	Rural	4 (67)	6 (75)	7 (100)	8 (89)	25 (83)
	Urban	2 (33)	2 (25)	—	1 (11)	5 (17)
**NCCN^d^**						
	Yes	2 (33)	2 (25)	2 (29)	3 (33)	9 (30)
	No or unsure	3 (50)	3 (38)	5 (71)	6 (67)	17 (57)
	N/A^e^	1 (17)	3 (38)	—	—	4 (13)
**Display mode**						
	Light	2 (33)	3 (38)	4 (57)	7 (78)	16 (53)
	Dark	4 (67)	5 (63)	3 (43)	2 (22)	14 (47)
**Mobile**						
	No mobile device	3 (50)	4 (50)	5 (71)	6 (67)	18 (60)
	Basic phone	—	—	—	1 (11)	1 (3)
	Android	2 (33)	2 (25)	—	—	4 (13)
	Apple	1 (17)	2 (25)	2 (29)	2 (22)	7 (23)
**Oncologist**						
	Lung, head, and neck	6 (100)	6 (75)	6 (86)	7 (78)	25 (83)
	Bone and soft tissue sarcomas, colorectal, pancreatic, and hepatobiliary	—	2 (25)	1 (14)	2 (22)	5 (17)

^a^Some percentages may not add up to 100 due to rounding.

^b^UI: user interface.

^c^Not available.

^d^NCCN: National Comprehensive Cancer Network.

^e^N/A: not applicable.

### Participant Demographics

The demographics of participants are summarized in Table 1. Most participants were older than 50 years (24/30, 80%), had up to a high school education (19/30, 63%), lived in a rural area (25/30, 83%), and were unfamiliar with mHealth apps (21/30, 70%). Participant sex and general mobile app use were split, with slightly more female participants (16/30, 53%) and users of mobile apps with a frequency of at least sometimes or more (17/30, 57%). Approximately half of the participants (16/30, 53%) used Assuage in light mode, and the rest (14/30, 47%) used Assuage in dark mode. While we did not gather specific data on each participant’s cancer type and mode of treatment, the 2 oncologists who were a part of this study specialize in the following: (1) all forms of lung, head, and neck cancers; and (2) bone and soft tissue sarcomas, colorectal, pancreatic, and hepatobiliary cancers. In total, 83% (25/30) of the participants were patients of the first oncologist, and 17% (5/30) were patients of the second oncologist. Most participants (29/30, 97%) resided in Kentucky, and the remainder (1/30, 3%) resided in West Virginia.

### System Usability Scores

**Figure 8:**
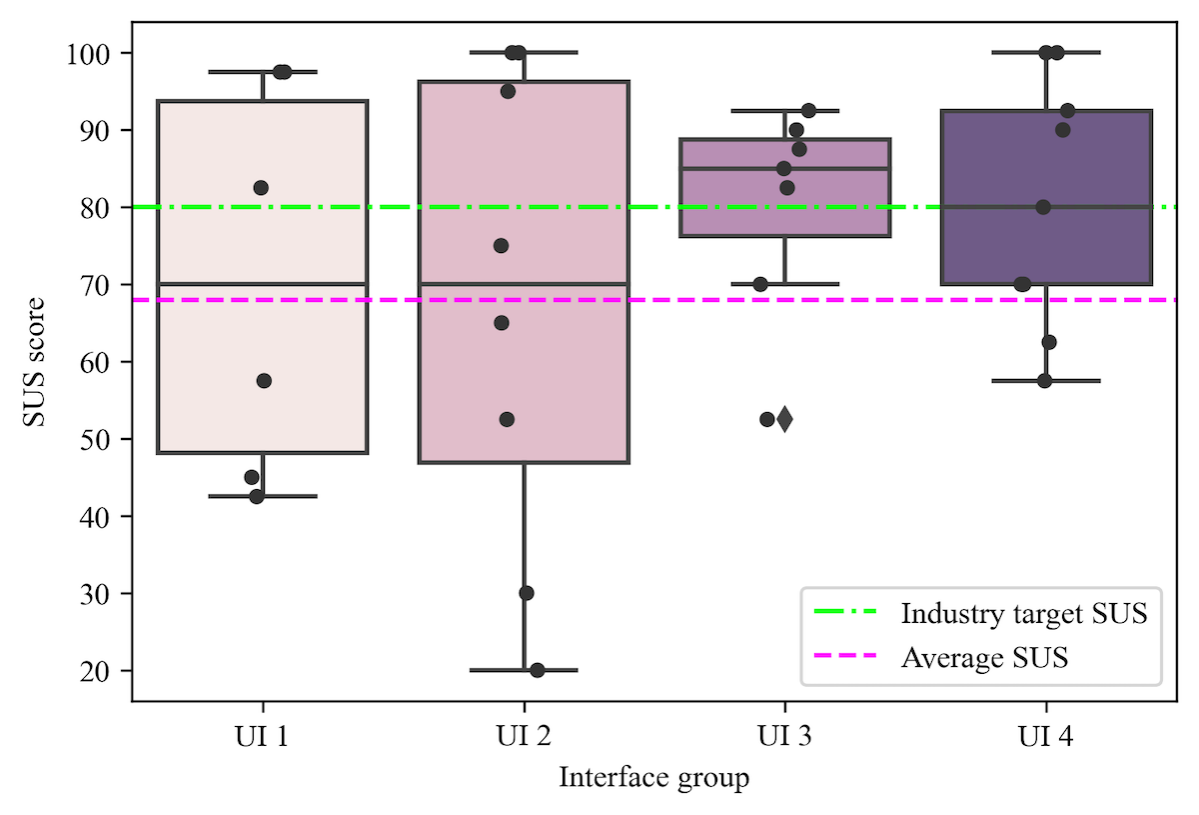
Boxplots depicting the distribution of System Usability Scale (SUS) scores grouped by the user interface (UI).

**Figure 9:**
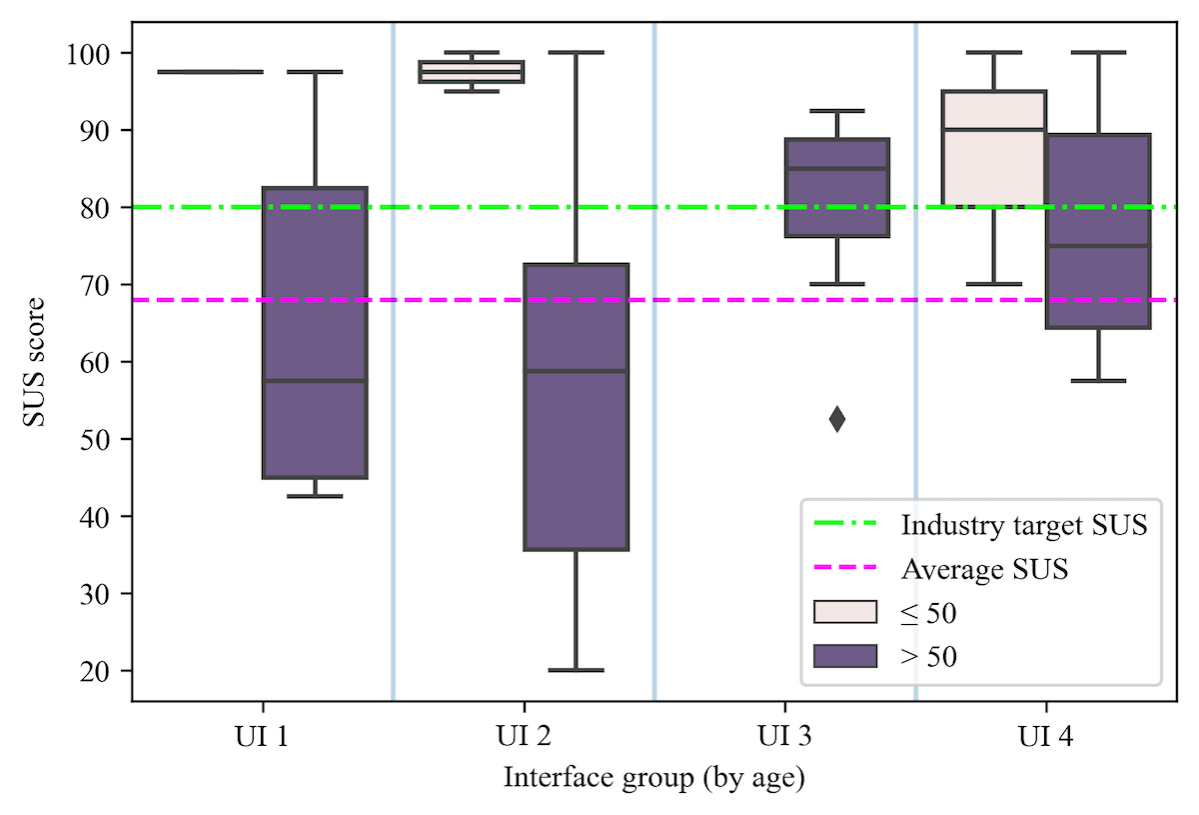
Boxplots depicting the distribution of System Usability Scale (SUS) scores grouped by the interface and age. UI: user interface.

The mean SUS score across the UIs was 75.8 (SD 22.2). Participants were randomly distributed across the 4 UIs. Among the 30 participants, 6 (20%) assessed UI 1 with a mean SUS score of 70.4 (SD 25.3), 8 (27%) assessed UI 2 with a mean SUS score of 67.2 (SD 31.2), 7 (23%) assessed UI 3 with a mean SUS score of 80.0 (SD 14.1), and 9 (30%) assessed UI 4 with a mean SUS score of 80.3 (SD 16.1). The SUS scores for each UI are reported in Table 2. Figure 8 shows the distribution of SUS scores for the different UI groups in relation to different target SUS scores. The dashed line represents an acceptable usability rating of ≥68 [81]. The dash-dotted line represents the industry target score of 80 to determine good usability [81]. Of the 4 UIs, 3 (UI 1, UI 3, and UI 4) had an average SUS score above the acceptable threshold of at least 68 and 2 (UI 3 and UI 4) met the industry threshold of at least 80. The average SUS score of UI 2 was just below what can be considered acceptable usability by 0.8 points. A one-way ANOVA was done to compare the effect of the UIs on the SUS scores. However, the results were not statistically significant (*F*_3,26_=0.68; *P*=.57). Figure 9 depicts the SUS scores across the UIs grouped by participant age. Additional figures depicting the SUS scores across the different UIs grouped by participant mobile use and light mode versus dark mode are shown in Figures S1 and S2 in Multimedia Appendix 1. The averages of these groupings are shown in Table S2 in Multimedia Appendix 1. The average score for each item on the SUS (Figure 2) is also reported in Table S3 in Multimedia Appendix 1. Of the participants who rated the UIs in Assuage as having less-than-acceptable usability, all were aged >50 years and unfamiliar with health apps (10/30, 33%) and a couple did not regularly use mobile apps (2/30, 7%).

**Table 2:** An overview of the usability for each user interface (UI) group.

Interface	Users (N=30), n (%)	SUS^a^ score, mean (SD)	SUS score, median (IQR)	Unacceptable usability, n (%)	Usability issues (N=36), n (%)
UI 1	6 (20)	70 (25)	70 (48-94)	3 (50)	1 (3)
UI 2	8 (27)	67 (31)	70 (47-96)	4 (50)	11 (31)
UI 3	7 (23)	80 (14)	85 (76-89)	1 (14)	14 (39)
UI 4	9 (30)	80 (16)	80 (70-93)	2 (22)	10 (28)

^a^SUS: System Usability Scale.

An overview of the usability for each UI group is presented in Table 2, including the number of participants per group, the mean and median SUS scores per group, how many participants rated a UI with a less-than-acceptable usability score, and how many usability issues occurred with each UI group.

Though our findings depict differences in scores between participant groups, they are not as significant. For example, UI 3 had a mean difference of approximately 14 points, and UI 4 had a mean difference of approximately 1.3 points when comparing mobile app users to nonusers (Table S2 in Multimedia Appendix 1). Similarly, when looking at participants aged >50 years, UI 3 and UI 4 have a smaller variance in usability score, and UI 3 had the tightest distribution with only 1 user rating below acceptable usability. Another interesting finding was the difference in usability scores among interfaces in dark mode and light mode. Aside from UI 1, the UIs in dark mode received significantly lower average usability scores, approximately 20 to 30 points, when compared to light mode.

### Usability Issues

Although all UIs were considered usable for patients, there were quite a few usability issues that could correlate with users’ lack of digital literacy. Approximately half of the participants (16/30, 53%) encountered usability issues when using Assuage. Most participants who experienced issues were aged >50 years (13/16, 81%) and did not regularly use mobile apps (12/16, 75%). A total of 16 usability problems were identified during the study. The usability issues experienced were divided into the following themes: data input and collection (15 issues), navigation (12 issues), instructions (3 issues), NCCN (4 issues), and color and interaction (2 issues). Table 3 presents the usability issues and the frequency of occurrence. Data input and collection are issues that could affect the user’s accuracy and input of distress data. Navigation issues are related to how the user navigates the assessments within the app. Instructions are issues where clearer instruction is needed. NCCN are issues related to the NCCN questionnaire. Color and interaction are usability issues that did not fit well in the previous themes.

**Table 3:** Usability issues experienced by users and frequency of occurrence.

Theme and usability issues			Frequency of occurrence (N=36), n (%)
**Data input and collection**			15 (42)
	Unclear about how to respond to the distress scale	7 (19)	
	Unclear how to indicate no to a specific symptom	3 (8)	
	Unclear what to do when no symptoms	2 (6)	
	Unsure if assessment was done and submitted	3 (8)	
**Navigation**			12 (33)
	Confusion when needing to vertical scroll	4 (11)	
	Uncertainty on how to start assessment	1 (3)	
	Unclear how to skip sections	3 (8)	
	Unsure how to continue to the next part of the assessment	2 (6)	
	Accidental navigation to other parts of the app	1 (3)	
	Tapping on the wrong button to complete surveys	1 (3)	
**Instructions**			3 (8)
	In-app instructions not clear	2 (6)	
	Review page unclear	1 (3)	
**NCCN^a^**			4 (11)
	Question wording confusing	3 (8)	
	Too many questions	1 (3)	
**Color and interaction**			2 (6)
	Confusion when the log button changed colors	1 (3)	
	Hard to take a pic of the paper form	1 (3)	

^a^NCCN: National Comprehensive Cancer Network.

### Participant Feedback

Patients had mixed perceptions of the different UIs’ learnability and usefulness. Positive responses from the participants described the UIs as easy, simple, intuitive, helpful, and good. Negative responses can be summarized as difficult, nonintuitive, inconsistent, and not for everyone. Regarding overall willingness to use an app for self-reporting symptoms, 7% (2/30) of the participants explicitly said they would want to use a symptom-reporting app more frequently (separate from the SUS item 1 [Figure 2], which states, “I think that I would be willing to use this system frequently”). Participants also expressed that if a physician told them to use the app, they would. Table 4 presents selected participants’ comments after using Assuage.

Desired features and improvements for reporting distress symptoms in an mHealth platform included distress data being sent directly to the physician, flagging the medical team if a patient reports high distress, prompts following completion of the distress assessment that can direct patients on who to contact depending on symptoms reported, proper feedback letting the patient know that their answers have been recorded, and an option to answer “none” if the patient has no symptoms instead of choosing to skip the question set.

**Table 4:** Selected participant comments following usability testing. Demographic data of the participants and the user interface (UI) they used are included.

Sentiment	Comments	UI
Positive	“Someone like me, if they know just a little stuff, then they’d be able to use it.” [Aged >50 years, high school education, does not use mobile apps]	UI 4
Mixed	It was not easy for this participant, but they did not feel it would be hard for others to learn. [Aged >50 years, high school education, does not use mobile apps]	UI 2
Negative	“Just doesn’t pertain to everybody.” [Aged >50 years, high school education, does not use mobile apps]	UI 2
Negative	“Not a lot of people computer savvy.” [Aged >50 years, high school education, uses mobile apps]	UI 4
Negative	“Would be difficult to older people.” [Aged >50 years, some college, uses mobile apps]	UI 2

## Discussion

### Principal Findings

This study assessed if state-of-the-art mobile app interface designs from Apple’s open-source ResearchKit and CareKit libraries would be usable for patients with cancer from rural areas. We leveraged the UI elements from Apple’s ResearchKit and CareKit frameworks to implement 4 different UI designs for patients to complete the NCCN distress assessment on the Assuage platform. The UIs varied by how the assessment questions were presented and navigated. This pilot study found that a survey-based app developed with Apple’s open-source libraries had a usable interface for patients with cancer within our target demographic. Specifically, using these frameworks, we achieved acceptable usability scores among nontraditional users, such as those who were older and did not regularly use mobile apps. The implication is that the frameworks are suitable for carrying out mHealth research with this demographic and can be used as a base for full-stack mHealth apps.

In addition, we evaluated if co-designing the interfaces was necessary to achieve acceptable usability with patients with cancer who were older or from rural areas. The results of this study show that it is possible to achieve good usability without co-design, which can reduce the time and resources spent in the design and development stages of a system or app for conducting mHealth research. Predictably, participants who were older than 50 years and did not use mobile devices regularly experienced the most usability issues. The most prominent usability issues were related to data input and navigation, with 15 and 12 occurrences, respectively. The most critical usability issues were participants needing to learn how to answer the distress scale and the UI assuming a participant knows when to scroll vertically. Not only did these 2 issues have the highest count of participants who experienced them, 23% (7/30) and 13% (4/30), respectively, but not addressing them can hinder participant completion of the survey, accurate reporting of symptoms and distress, and motivation to use the system.

Finally, we wanted to understand what caused a specific UI to have a higher usability rating than the others as a basis to move forward for future research studies with our target demographic. Our findings show that most participants (20/30, 67%) rated the UIs as having acceptable and above-average usability across the different interfaces, with UI 3 and UI 4 averaging approximately 10 points higher than UI 1 and UI 2 using the SUS. UI 3 and UI 4 also met the industry threshold for good usability with average SUS scores of at least 80. Despite navigation and input challenges, participants could still complete the in-app survey and expressed the willingness to use an mHealth system for self-reporting symptoms. Unsurprisingly, participants were more concerned about what happened after reporting symptoms, such as whether the physician would be notified or if the participant would receive feedback on how to continue based on the reported symptoms.

### Comparison With Prior Work

Prior work suggests that mHealth systems should be co-designed with target users for optimal outcomes and usability [7,8,40]. Aronoff-Spencer et al [40] used participatory design to recreate an alternative design to the NCCN assessment. Digital and paper prototypes of the redesigned survey were compared to the original using the SUS, resulting in patients finding the digital prototypes more usable than their paper counterparts. The usability of Assuage’s different UIs was comparable to the co-designed prototypes without undergoing the resource-intensive process. Similarly, our usability results were comparable to other mHealth studies using the SUS to assess iterative designs [25,102-104].

While the usability issues encountered by participants could be attributed to digital literacy, developers can take extra steps to ensure universal design when using development frameworks. Formatting a survey for web and mobile delivery has been evaluated, but has had conflicting results [65-67,69]. For example, usability heuristics say that vertical and horizontal scrolling should be avoided when possible. Apple’s Human Interface Guidelines provide best practices for scroll views, including the use of scroll indicators. These indicators show users how much of the content they have scrolled through and how much is left [97]. Designing using paging instead of scrolling formats surveys in a clean and easy-to-read manner. Minimizing scrolling prevents users from missing questions or important interface elements, such as navigation buttons. Alternatively, studies have also found that scrolling layouts resulted in higher perceived usability and faster survey completion times [65,66]. Our usability results were slightly better with a paging design (UI 2 vs UI 3, with an average usability score of 67.2, SD 31.2 and 80.0, SD 14.1, respectively). UI 4 used Apple’s CareKit UI (a modular design combined with vertical scrolling) and received good usability scores (mean 80.3, SD 16.1), contradicting some of the best practices found in the literature. Notably, the modularized surveys are displayed in a manner similar to paging designs. In addition, it is interesting to note that the 2 UIs that provided more freedom in navigating the survey were the most highly rated. Reflecting on the usability heuristics by Nielsen (Figure 7), the navigation schemes implemented in UI 3 and UI 4 were the only interfaces that satisfied the heuristic (#7) of flexibility and efficiency of use. Considering the visual similarities between UI 2 and UI 3, we can infer that the flexible navigation, coupled with the grouping of questions on different pages, significantly improved usability scores.

Prior work suggests that respondents should be offered a “none” option or similar when presented with a list of other choices [68]. However, the placement of that option influences whether participants choose it. Placing an option, such as “none,” when other choices do not apply at the top of the page results in more respondents choosing it compared to when placed at the bottom of the survey [67], which can be important to consider for the thoroughness of data. In our case, we did not require participants to input an answer in every section and included a “skip” option at the bottom of the page, separate from the possible symptom choices. Nevertheless, some participants would have preferred an actual answer choice instead of skipping the page, as it made them feel like they were not fully completing the assessment. At times, the “skip” button did not stand out to participants as a tappable button compared to the “next” button, which had a visible background (eg, Figure 5, steps 2-3).

Participants encountered the most problems with the distress scale. The use of rating scales in surveys is fairly common [29,30]; however, for some participants, it was not intuitive to slide or tap to interact with the distress scale. All but one of the participants (6/7, 86%) who experienced this problem did not regularly use mobile apps. We attempted to keep the question format as similar to the original NCCN assessment as possible; however, an alternative format to a rating scale could be a number picker or text entry with specific number values. Similar to the symptoms, a list view could also be considered, although potentially less efficient if all numbers do not fit on the device screen. Alternatively, gestural signifiers can be used to demonstrate how to complete tasks. The findings of this usability study support prior research on electronic survey design, particularly with aging users, such as those older than 50 years, which should be considered when using frameworks that provide predetermined UI features. It should be noted that although important, prior work suggests that question wording does not affect usability as much as the layout [67].

Regarding the use of dark mode versus light mode in UI designs, studies have investigated how the trend of dark mode, or negative polarity, interfaces impact users [105-108]. A recent study found that light-mode interfaces are more advantageous to young and older users concerning cognitive load [106]. Considering most of our participants were aged >50 years, this could give insight into the drastic difference in usability scores between those who used Assuage in light mode and those who used Assuage in dark mode. Similarly, many patients with cancer and survivors of cancer experience cognitive effects due to cancer and its treatment [39]. Therefore, while developers of mHealth systems can implement a dark-mode interface, they must ensure that the different UI elements do not create unnecessary cognitive burden for users [107]. However, based on these preliminary results, not implementing dark mode should not have an adverse effect on our demographic of patients with cancer who are older than 50 years and from rural areas.

### Limitations and Future Work

A sample size of 30 is typically considered small; however, previous research on system usability studies implies that small sample sizes, approximately 5, can capture most usability issues [85,86]. This study was also interrupted due to a spike in COVID-19 cases, which resulted in the hospital halting all nonessential and nonmedical activities, limiting our sample size. We attempted to use additional techniques during usability testing, such as a think-aloud approach; however, as patient participants were being seen between appointments, brain fog from chemotherapy treatments resulted in frustration from participants with this approach. Excluding cognitive impairment due to cancer-related treatments, the normal aging process can also cause a decline in cognitive function for older people in similar studies. Similarly, with respect to participant time, the study survey was kept as short as possible. This further supported our choice to use the SUS versus a more in-depth questionnaire, such as the Mobile Application Rating Scale [109], the Health Information Technology Usability Evaluation Scale [110], or the mHealth App Usability Questionnaire [111]. Finally, we invited health care professionals to assess Assuage; however, only 1 responded, and we did not include their SUS score in this paper.

Despite limitations, we identified areas of improvement for the interface design of surveys in mobile apps. We also determined which UIs in Assuage would be suitable for future deployment studies with our target demographic of patients with cancer who are from rural areas, older than 50 years, and may not regularly use mobile apps. Not all participants owned mobile devices, posing a potential wide-scale implementation problem. While reports show smartphone use to be consistently rising among members of the rural United States, this may not be consistent across all rural areas. Conversely, participants without mobile devices usually had other family members with mobile devices and smartphones. Most participants expressed a willingness to use an app to monitor their symptoms. However, deploying the app among rural patients in the southeastern and Appalachian regions is necessary to determine if apps are a viable solution for this demographic. In the future, we plan to conduct follow-up studies to assess adherence and reasons for engagement with Assuage to report distress symptoms of patients over time.

### Conclusions

Digital implementations of validated paper-based surveys can have unexpected outcomes on the usability of the survey and an app. If a digital survey has low usability, patients could be deterred from entering information, or the data could be unreliable, limiting the tool’s effectiveness. This could also affect research findings from this method or how the clinic responds. The findings show that 67% (20/30) of the patients with cancer who participated in this pilot usability study rated the different interfaces of Assuage as above average (SUS score >68) [81]. This suggests that Apple’s health and research frameworks provide usable UIs with minimal alterations to the default interface for users older than 50 years and with limited digital literacy. The usability issues observed align with common usability problems for designing surveys. ResearchKit and CareKit can be used to reliably design a mobile app for collecting survey-based data. However, heuristics for both usability and electronic survey design should be considered when deciding how to best segment and navigate surveys and how to present important interface elements.

The main difference between the UIs was how users could navigate between the different survey sections. The interfaces that satisfied the heuristic by Nielsen regarding flexibility and efficiency of use (#7), allowed users to freely jump between survey sections nonsequentially and achieved the highest usability scores. Therefore, it can be inferred that flexible question navigation is a feature that should not be overlooked when digitizing surveys. Other ways to increase the usability of interface designs for self-reporting outcomes by patients who do not frequently use mobile apps include gestural signifiers; visual cues when scrolling is available, such as scroll indicators; minimizing scrolling per page; and a dedicated answer choice when none apply.

The findings from this paper do not aim to undermine the importance or benefits of co-design or participatory design for underserved and understudied populations but to demonstrate the possibility for successful digital implementations when resources cannot be heavily allocated to the design process. Although the UIs in the Assuage app had overall good usability, if resources and time permit, involving end users in the design process can improve the overall usability of the final product, increasing the chance for sustained use. However, for survey-based mHealth iOS apps, ResearchKit and CareKit are legitimate options for developers and researchers seeking open-source libraries with suitable interface designs to use with similar populations to this study. Participatory design is still suggested to understand key features to support users unfamiliar with smart devices and touch interfaces when assistance is not readily available. A follow-up longitudinal study deploying Assuage with end users is currently underway.

## Appendix

Multimedia Appendix 1 Additional tables and figures related to the interface design and results.

## Abbreviations

HIPAA: Health Insurance Portability and Accountability Act
mHealth: mobile health
NCCN: National Comprehensive Cancer Network
SUS: System Usability Scale
UI: user interface

## References

[1] Jiang Y, West BT, Barton DL, Harris MR (2017). Acceptance and use of eHealth/mHealth applications for self-management among cancer survivors. Stud Health Technol Inform.

[2] Potdar R, Thomas A, DiMeglio M, Mohiuddin K, Djibo DA, Laudanski K, Dourado CM, Leighton JC, Ford JG (2020). Access to internet, smartphone usage, and acceptability of mobile health technology among cancer patients. Support Care Cancer.

[3] Fareed N, Swoboda CM, Jonnalagadda P, Huerta TR (2021). Persistent digital divide in health-related internet use among cancer survivors: findings from the Health Information National Trends Survey, 2003-2018. J Cancer Surviv.

[4] Underserved group. U.S. Department of Health and Human Services, National Institutes of Health, National Center for Advancing Translational Sciences.

[5] Hartch CE, Dietrich MS, Stolldorf DP (2023). Effect of a medication adherence mobile phone app on medically underserved patients with chronic illness: preliminary efficacy study. JMIR Form Res.

[6] Kent EE, Lee S, Asad S, Dobbins EE, Aimone EV, Park EM (2023). "If I wasn't in a rural area, I would definitely have more support": social needs identified by rural cancer caregivers and hospital staff. J Psychosoc Oncol.

[7] Hesse BW, Ahern D, Ellison M, Aronoff-Spencer E, Vanderpool RC, Onyeije K, Gibbons MC, Mullett TW, Chih MY, Attencio V, Patterson G, Boten J, Hartshorn C, Bartolome B, Gorscak K, McComsey M, Hubenko A, Huang B, Baker C, Norman D (2020). Barn-raising on the digital frontier: the L.A.U.N.C.H. collaborative. J Appalach Health.

[8] McComsey M, Ahern D, Vanderpool RC, Mullett TW, Chih MY, Johnson M, Ellison M, Onyeije K, Hesse BW, Aronoff-Spencer E (2020). Experiencing cancer in Appalachian Kentucky. J Appalach Health.

[9] Morris BB, Hughes R, Fields EC, Sabo RT, Weaver KE, Fuemmeler BF (2023). Sociodemographic and clinical factors associated with radiation treatment nonadherence and survival among rural and nonrural patients with cancer. Int J Radiat Oncol Biol Phys.

[10] Sepassi A, Li M, Zell JA, Chan A, Saunders IM, Mukamel DB (2024). Rural-urban disparities in colorectal cancer screening, diagnosis, treatment, and survivorship care: a systematic review and meta-analysis. Oncologist.

[11] Faber JS, Al-Dhahir I, Kraal JJ, Breeman LD, van den Berg-Emons RJ, Reijnders T, van Dijk S, Janssen VR, Kraaijenhagen RA, Visch VT, Chavannes NH, Evers AW (2023). Guide development for eHealth interventions targeting people with a low socioeconomic position: participatory design approach. J Med Internet Res.

[12] Eba K, Gerbaba MJ, Abera Y, Tadessse D, Tsegaye S, Abrar M, Mohammed A, Ibrahim A, Shekabdulahi M, Zeleke S, Medhin G (2023). Mobile health service as an alternative modality for hard-to-reach pastoralist communities of Afar and Somali regions in Ethiopia. Pastoralism.

[13] Stiles-Shields C, Reyes KM, Archer J, Lennan N, Zhang J, Julion WA, Karnik NS (2022). mHealth uses and opportunities for teens from communities with high health disparities: a mixed-methods study. J Technol Behav Sci.

[14] Schreurs L, Steenhout I, Bosmans J, Buyl R, De Cock D (2023). Can mHealth bridge the digital divide in rheumatic and musculoskeletal conditions?. BMC Digit Health.

[15] Taramasco C, Rimassa C, Noël R, Bravo Storm ML, Sánchez C (2023). Co-design of a mobile app for engaging breast cancer patients in reporting health experiences: qualitative case study. J Med Internet Res.

[16] Grove BE, de Thurah A, Ivarsen P, Kvisgaard AK, Hjollund NH, Grytnes R, Schougaard LM (2024). Remote symptom monitoring using patient-reported outcomes in patients with chronic kidney disease: process evaluation of a randomized controlled trial. JMIR Form Res.

[17] Gelles-Watnick R (2024). Americans’ use of mobile technology and home broadband. Pew Research Center.

[18] (2024). Mobile fact sheet. Pew Research Center.

[19] Edwards ER, Fei-Zhang DJ, Stein AP, Lott DG, Chelius DC, Sheyn A, Rastatter J (2024). The impact of digital inequities on laryngeal cancer disparities in the US. Am J Otolaryngol.

[20] Reddick C, Enriquez R, Harris R, Flores J (2024). Understanding the levels of digital inequality within the city: an analysis of a survey. Cities.

[21] Peck EM, Ayuso SE, El-Etr O (2019). Data is personal: attitudes and perceptions of data visualization in rural Pennsylvania. Proceedings of the 2019 CHI Conference on Human Factors in Computing Systems.

[22] Anthony DL, Campos-Castillo C, Lim PS (2018). Who isn't using patient portals and why? Evidence and implications from a national sample of US adults. Health Aff (Millwood).

[23] Zhang Y, Xu P, Sun Q, Baral S, Xi L, Wang D (2023). Factors influencing the e-health literacy in cancer patients: a systematic review. J Cancer Surviv.

[24] Deniz-Garcia A, Fabelo H, Rodriguez-Almeida AJ, Zamora-Zamorano G, Castro-Fernandez M, Alberiche Ruano MD, Solvoll T, Granja C, Schopf TR, Callico GM, Soguero-Ruiz C, Wägner AM (2023). Quality, usability, and effectiveness of mHealth apps and the role of artificial intelligence: current scenario and challenges. J Med Internet Res.

[25] Alqahtani F, Alslaity A, Orji R (2022). Usability testing of a gratitude application for promoting mental well-being. Proceedings of the Human-Computer Interaction. User Experience and Behavior.

[26] Baldwin JL, Singh H, Sittig DF, Giardina TD (2017). Patient portals and health apps: pitfalls, promises, and what one might learn from the other. Healthc (Amst).

[27] Teles S, Paúl C, Lima P, Chilro R, Ferreira A (2021). User feedback and usability testing of an online training and support program for dementia carers. Internet Interv.

[28] Oppenheimer AJ, Pannucci CJ, Kasten SJ, Haase SC (2011). Survey says? A primer on web-based survey design and distribution. Plast Reconstr Surg.

[29] Nayak MS, Narayan KA (2019). Strengths and weaknesses of online surveys. IOSR J Humanit Soc Sci.

[30] Maymone MB, Venkatesh S, Secemsky E, Reddy K, Vashi NA (2018). Research techniques made simple: web-based survey research in dermatology: conduct and applications. J Invest Dermatol.

[31] Davis FD (1989). Perceived usefulness, perceived ease of use, and user acceptance of information technology. MIS Q.

[32] Bolaños M, Collazos C, Gutiérrez F (2021). Experiences in the application of some models of technology acceptance: adaptation for the elderly people. Proceedings of the XXI International Conference on Human Computer Interaction.

[33] Meiryani M, Chang A, Lorenzo BA, Daud Z (2021). Analysis of technology acceptance model (TAM) approach to the quality of accounting information systems. Proceedings of the 9th International Conference on Computer and Communications Management.

[34] Ding EY, Pathiravasan CH, Schramm E, Borrelli B, Liu C, Trinquart L, Kornej J, Benjamin EJ, Murabito JM, McManus DD (2021). Design, deployment, and usability of a mobile system for cardiovascular health monitoring within the electronic Framingham Heart Study. Cardiovasc Digit Health J.

[35] Gance-Cleveland B, Leiferman J, Aldrich H, Nodine P, Anderson J, Nacht A, Martin J, Carrington S, Ozkaynak M (2019). Using the technology acceptance model to develop StartSmart: mHealth for screening, brief intervention, and referral for risk and protective factors in pregnancy. J Midwifery Womens Health.

[36] Holden RJ, Karsh BT (2010). The technology acceptance model: its past and its future in health care. J Biomed Inform.

[37] Harrington CN, Ruzic L, Sanford JA (2017). Universally accessible mHealth apps for older adults: towards increasing adoption and sustained engagement. Proceedings of the 11th International Conference on Universal Access in Human–Computer Interaction. Human and Technological Environments.

[38] Nicol E, Dunlop M, Komninos A, McGee-Lennon M, Baillie L, Edwards A, Eslambolchilar P, Goodman-Deane J, Hakobyan L, Lumsden J, Mulder I, Rau P, Siek K (2014). Re-imagining commonly used mobile interfaces for older adults. Proceedings of the 16th International Conference on Human-Computer Interaction With Mobile Devices & Services.

[39] Adler RF, Morales P, Sotelo J, Magasi S (2022). Developing an mHealth app for empowering cancer survivors with disabilities: co-design study. JMIR Form Res.

[40] Aronoff-Spencer E, McComsey M, Chih MY, Hubenko A, Baker C, Kim J, Ahern DK, Gibbons MC, Cafazzo JA, Nyakairu P, Vanderpool RC, Mullett TW, Hesse BW (2022). Designing a framework for remote cancer care through community co-design: participatory development study. J Med Internet Res.

[41] Distress. National Institutes of Health National Cancer Institute.

[42] Ownby KK (2019). Use of the distress thermometer in clinical practice. J Adv Pract Oncol.

[43] Smith SK, Loscalzo M, Mayer C, Rosenstein DL (2018). Best practices in oncology distress management: beyond the screen. Am Soc Clin Oncol Educ Book.

[44] Gessler S, Low J, Daniells E, Williams R, Brough V, Tookman A, Jones L (2008). Screening for distress in cancer patients: is the distress thermometer a valid measure in the UK and does it measure change over time? A prospective validation study. Psychooncology.

[45] Albrecht TA, Rosenzweig M (2012). Management of cancer-related distress in patients with a hematologic malignancy. J Hosp Palliat Nurs.

[46] Meilleur A, Subramanian SV, Plascak JJ, Fisher JL, Paskett ED, Lamont EB (2013). Rural residence and cancer outcomes in the United States: issues and challenges. Cancer Epidemiol Biomarkers Prev.

[47] (2023). NCCN guidelines version 1.2024: distress management. National Comprehensive Cancer Network.

[48] Vitek L, Rosenzweig M, Stollings S (2007). Distress in patients with cancer: definition, assessment, and suggested interventions. Clin J Oncol Nurs.

[49] Zebrack B, Kayser K, Bybee D, Padgett L, Sundstrom L, Jobin C, Oktay J (2017). A practice-based evaluation of distress screening protocol adherence and medical service utilization. J Natl Compr Canc Netw.

[50] Jacobs M, Hopkins J, Mumber M, Mynatt E (2019). Usability evaluation of an adaptive information recommendation system for breast cancer patients. AMIA Annu Symp Proc.

[51] van Acker J, Maenhout L, Compernolle S (2023). Older adults' user engagement with mobile health: a systematic review of qualitative and mixed-methods studies. Innov Aging.

[52] Abahussin AA, West RM, Wong DC, Ziegler LE, Allsop MJ (2023). Supporting pain self-management in patients with cancer: app development based on a theoretical and evidence-driven approach. JMIR Cancer.

[53] Jim HS, Hoogland AI, Brownstein NC, Barata A, Dicker AP, Knoop H, Gonzalez BD, Perkins R, Rollison D, Gilbert SM, Nanda R, Berglund A, Mitchell R, Johnstone PA (2020). Innovations in research and clinical care using patient-generated health data. CA Cancer J Clin.

[54] Mitzner TL, Rogers WA, Fisk AD, Boot WR, Charness N, Czaja SJ, Sharit J (2016). Predicting older adults' perceptions about a computer system designed for seniors. Univers Access Inf Soc.

[55] Rogers ME, Rogers NL, Takeshima N, Islam MM (2003). Methods to assess and improve the physical parameters associated with fall risk in older adults. Prev Med.

[56] Introducing ResearchKit. ResearchKit.

[57] Powell MR, To WJ (2016). Redesigning the research design: accelerating the pace of research through technology innovation. Proceedings of the IEEE International Conference on Serious Games and Applications for Health.

[58] Berkowitz CM, Zullig LL, Koontz BF, Smith SK (2017). Prescribing an app? Oncology providers' views on mobile health apps for cancer care. JCO Clin Cancer Inform.

[59] Genes N, Violante S, Cetrangol C, Rogers L, Schadt EE, Chan YF (2018). From smartphone to EHR: a case report on integrating patient-generated health data. NPJ Digit Med.

[60] Overview - ResearchKit and CareKit. Apple Research & Care.

[61] van Gelder MM, Engelen LJ, Sondag T, van de Belt TH (2018). Utilizing consumer technology (Apple's ResearchKit) for medical studies by patients and researchers: proof of concept of the novel platform REach. J Particip Med.

[62] Bender D, Sartipi K (2013). HL7 FHIR: an agile and RESTful approach to healthcare information exchange. Proceedings of the 26th IEEE International Symposium on Computer-Based Medical Systems.

[63] Ensari I, Elhadad N, Syed-Abdul S, Zhu X, Fernandez-Luque L (2021). Chapter 5 - mHealth for research: participatory research applications to gain disease insights. Digital Health: Mobile and Wearable Devices for Participatory Health Applications.

[64] Bührmann L, Van Daele T, Rinn A, De Witte NA, Lehr D, Aardoom JJ, Loheide-Niesmann L, Smit J, Riper H (2022). The feasibility of using Apple's ResearchKit for recruitment and data collection: considerations for mental health research. Front Digit Health.

[65] Shannon DM, Johnson TE, Searcy S, Lott A (2002). Using electronic surveys: advice from survey professionals. Pract Assess Res Eval.

[66] Marcano Belisario JS, Jamsek J, Huckvale K, O'Donoghue J, Morrison CP, Car J (2015). Comparison of self-administered survey questionnaire responses collected using mobile apps versus other methods. Cochrane Database Syst Rev.

[67] Couper MP (2016). Usability evaluation of computer-assisted survey instruments. Soc Sci Comput Rev.

[68] Ball HL (2019). Conducting online surveys. J Hum Lact.

[69] Chambers S, Nimon K, Anthony-McMann P (2016). A primer for conducting survey research using MTurk: tips for the field. Int J Adult Vocat Educ Technol.

[70] Nielsen J (1994). 10 Usability heuristics for user interface design. Nielsen Norman Group.

[71] Nielsen J (1994). Enhancing the explanatory power of usability heuristics. Proceedings of the SIGCHI Conference on Human Factors in Computing Systems.

[72] Benze G, Nauck F, Alt-Epping B, Gianni G, Bauknecht T, Ettl J, Munte A, Kretzschmar L, Gaertner J (2019). PROutine: a feasibility study assessing surveillance of electronic patient reported outcomes and adherence via smartphone app in advanced cancer. Ann Palliat Med.

[73] Norman DA, Stappers PJ (2015). DesignX: complex sociotechnical systems. She Ji.

[74] Kirkscey R (2020). mHealth apps for older adults: a method for development and user experience design evaluation. J Tech Writ Commun.

[75] Haines ER, Dopp A, Lyon AR, Witteman HO, Bender M, Vaisson G, Hitch D, Birken S (2021). Harmonizing evidence-based practice, implementation context, and implementation strategies with user-centered design: a case example in young adult cancer care. Implement Sci Commun.

[76] Hardy J, Wyche S, Veinot T (2019). Rural HCI research: definitions, distinctions, methods, and opportunities. Proc ACM Hum Comput Interact.

[77] Stowell E, Lyson MC, Saksono H, Wurth RC, Jimison J, Pavel M, Parker AG (2018). Designing and evaluating mHealth interventions for vulnerable populations: a systematic review. Proceedings of the 2018 CHI Conference on Human Factors in Computing Systems.

[78] Kohavi R, Longbotham R, Sammut C, Webb GI (2017). Online controlled experiments and A/B testing. Encyclopedia of Machine Learning and Data Mining.

[79] Romero OJ, Haig A, Kirabo L, Yang Q, Zimmerman J, Tomasic A, Steinfeld A (2020). A long-term evaluation of adaptive interface design for mobile transit information. Proceedings of the 22nd International Conference on Human-Computer Interaction with Mobile Devices and Services.

[80] Brooke J, Jordan PW, Thomas B, McClelland IL, Weerdmeester B (1996). SUS: a 'quick and dirty' usability scale. Usability Evaluation In Industry.

[81] Lewis JR, Sauro J (2018). Item benchmarks for the system usability scale. J Usability Stud.

[82] Orfanou K, Tselios N, Katsanos C (2015). Perceived usability evaluation of learning management systems: empirical evaluation of the System Usability Scale. Int Rev Res Open Distrib Learn.

[83] Hyzy M, Bond R, Mulvenna M, Bai L, Dix A, Leigh S, Hunt S (2022). System usability scale benchmarking for digital health apps: meta-analysis. JMIR Mhealth Uhealth.

[84] Lewis JR, Sauro J (2009). The factor structure of the system usability scale. Proceedings of the Human Centered Design.

[85] Fox JE (2015). The science of usability testing. Proceedings of the 2015 Federal Committee on Statistical Methodology (FCSM) Research Conference.

[86] Virzi RA (2016). Refining the test phase of usability evaluation: how many subjects is enough?. Hum Factors.

[87] Adesina N, Dogan H, Green S, Tsofliou F (2021). Effectiveness and usability of digital tools to support dietary self-management of gestational diabetes mellitus: a systematic review. Nutrients.

[88] Islam MN, Karim MM, Inan TT, Islam AK (2020). Investigating usability of mobile health applications in Bangladesh. BMC Med Inform Decis Mak.

[89] Resnick D, Kearney MD, Smith JM, Bautista A, Jones L, Schapira MM, Aysola J (2022). Designing a cancer prevention collaborative goal-setting mobile app for non-Hispanic Black primary care patients: an iterative, qualitative patient-led process. JMIR Form Res.

[90] Kluyver T, Ragan-Kelley B, Pérez F, Granger B, Bussonnier M, Frederic J, Kelley K, Hamrick J, Grout J, Corlay S, Ivanov P, Avila D, Abdalla S, Willing C, Loizides F, Schmidt B (2016). Jupyter Notebooks—a publishing format for reproducible computational workflows. Positioning and Power in Academic Publishing: Players, Agents and Agendas.

[91] Krippendorff K (1980). Content Analysis: An Introduction to Its Methodology.

[92] Rampin R, Rampin V (2021). Taguette: open-source qualitative data analysis. J Open Source Softw.

[93] HealthKit | Apple Developer Documentation. Apple Inc.

[94] netreconlab / ParseCareKit. GitHub.

[95] netreconlab / parse-hipaa. GitHub.

[96] Leader AE, Capparella LM, Waldman LB, Cammy RB, Petok AR, Dean R, Shimada A, Yocavitch L, Rising KL, Garber GD, Worster B, Dicker AP (2021). Digital literacy at an urban cancer center: implications for technology use and vulnerable patients. JCO Clin Cancer Inform.

[97] Human interface guidelines. Apple Inc.

[98] Ahmad FA, Payne PR, Lackey I, Komeshak R, Kenney K, Magnusen B, Metts C, Bailey T (2020). Using REDCap and Apple ResearchKit to integrate patient questionnaires and clinical decision support into the electronic health record to improve sexually transmitted infection testing in the emergency department. J Am Med Inform Assoc.

[99] Bent B, Goldstein BA, Kibbe WA, Dunn JP (2020). Investigating sources of inaccuracy in wearable optical heart rate sensors. NPJ Digit Med.

[100] Powers R, Etezadi-Amoli M, Arnold EM, Kianian S, Mance I, Gibiansky M, Trietsch D, Alvarado AS, Kretlow JD, Herrington TM, Brillman S, Huang N, Lin PT, Pham HA, Ullal AV (2021). Smartwatch inertial sensors continuously monitor real-world motor fluctuations in Parkinson's disease. Sci Transl Med.

[101] Lalloo C, Pham Q, Cafazzo J, Stephenson E, Stinson J (2020). A ResearchKit app to deliver paediatric electronic consent: protocol of an observational study in adolescents with arthritis. Contemp Clin Trials Commun.

[102] Hsieh KL, Fanning JT, Rogers WA, Wood TA, Sosnoff JJ (2018). A fall risk mHealth app for older adults: development and usability study. JMIR Aging.

[103] Teo CH, Ng CJ, Lo SK, Lim CD, White A (2019). A mobile web app to improve health screening uptake in men (ScreenMen): utility and usability evaluation study. JMIR Mhealth Uhealth.

[104] Ehrler F, Lovis C, Blondon K (2019). A mobile phone app for bedside nursing care: design and development using an adapted software development life cycle model. JMIR Mhealth Uhealth.

[105] Eisfeld H, Kristallovich F (2020). The rise of dark mode: a qualitative study of an emerging user interface design trend. Digitala Vetenskapliga Arkivet.

[106] Sethi T, Ziat M (2023). Dark mode vogue: do light-on-dark displays have measurable benefits to users?. Ergonomics.

[107] Andrew S, Bishop C, Tigwell GW (2024). Light and dark mode: a comparison between android and iOS app UI modes and interviews with app designers and developers. Proc ACM Interact Mob Wearable Ubiquitous Technol.

[108] Virtanen J (2023). Dark mode preferences: exploring user motivations in interface theme selection. University of Turku.

[109] Stoyanov SR, Hides L, Kavanagh DJ, Wilson H (2016). Development and validation of the user version of the Mobile Application Rating Scale (uMARS). JMIR Mhealth Uhealth.

[110] Schnall R, Cho H, Liu J (2018). Health information technology usability evaluation scale (Health-ITUES) for usability assessment of mobile health technology: validation study. JMIR Mhealth Uhealth.

[111] Zhou L, Bao J, Setiawan IM, Saptono A, Parmanto B (2019). The mHealth App Usability Questionnaire (MAUQ): development and validation study. JMIR Mhealth Uhealth.

