# Supplementary material for: Designing Survey-Based Mobile Interfaces for Rural Patients With Cancer Using Apple’s ResearchKit and CareKit: Usability Study
Source: JMIR Form Res. 2024 Sep 26;8:e57801. doi: 10.2196/57801 (PMC11467601; doi:10.2196/57801)

Table S1. The 10 usability heuristics by Nielsen and descriptions of how they were implemented within the different user interfaces (UI) in Assuage.

Nielsen's heuristics	Assuage implementation
(1) Visibility of system status	• UI 1 – N/A<br>• UI 2 – Displays page numbering referring to number of screens in the assessment<br>• UI 3 – Highlights the current section of the assessment<br>• UI 4 – Displays all possible sections on main screen and has a progress ring
(2) Match between system and real world	• All UIs – Survey format and questions matched to NCCN assessment<br>• UI 2, 3, and 4 – Scale format similar to a thermometer, and color gradient similar to how a thermometer may represent temperature
(3) User control and freedom	• UI 1 – Next and back buttons. Can retake picture before submitting.<br>• UI 2 – Next, back, and skip buttons. Can edit answers before submitting.<br>• UI 3 – Next, back, and skip buttons. Tappable buttons at top of interface to select assessment sections. Can edit answers before submitting.<br>• UI 4 – Can freely select which assessment section to complete. Cancel and skip buttons.
(4) Consistency and standards	• All UIs – Language mimicked NCCN assessment. Consistent use of color through out system. Interface elements consistent with other Apple products.
(5) Error prevention	• UI 1 – Can check picture and re-take before submitting.<br>• UI 2 and UI 3 – Can undo answers. Must select a symptom before the Next button can be tapped. Use of color to show whether a button can be tapped or not. Review screen before submitting answers.<br>• UI 4 – N/A
(6) Recognition rather than recall	• UI 1 – N/A<br>• UI 2, 3, and 4 – Each NCCN segment was clearly labeled. Each screen repeated instructions.
(7) Flexibility and efficiency of use	• UI 1 – N/A<br>• UI 2 – N/A<br>• UI 3 – Navigation segments at top of screen which allows for skipping to relevant sections.

	• UI 4 – NCCN assessment categories separated on a dashboard and users can select specific sections if they would like to respond.
(8) Aesthetic and minimalist design	• All UIs – Interface elements limited to what is necessary to complete the assessment. All buttons are for navigation and all words for instructions and survey options. Limited use of color so as not to be distracting.
(9) Recognize, diagnose, and recover from errors	• All UIs – N/A<br>• The UIs did not reflect this heuristic. However, due to the nature of the NCCN assessment, which limited possible responses to a number scale and binary (yes/no) options, users could not have input errors such as entering incorrect or out-of-range values.
(10) Help and documentation	• All UIs – Instructions on how to use Assuage to complete the NCCN assessment.<br>• UI 4 – Additional documentation describing the different symptom categories.

Table S2. Average System Usability Scale (SUS) scores for each user interface (UI) grouped by the age, mobile app use, and display mode.

**Variable**		**User interface (UI)**									
		**UI 1**		**UI 2**		**UI 3**		**UI 4**		Total	
		mean (SD)	n (%) n=6	mean (SD)	n (%) n=8	mean (SD)	n (%) n=7	mean (SD)	n (%) n=9	mean (SD)	n (%) N=30
**Age**											
	≤ 50	98	1 (17)	98 (4)	2 (25)	—[a]	—	87 (15)	3 (33)	92 (11)	6 (20)
	> 50	65 (24)	5 (83)	57 (30)	6 (75)	80 (14)	7 (100)	77 (17)	6 (67)	70 (22)	24 (80)
**Mobile app use**											
	No	48 (8)	3 (50)	34 (17)	3 (38)	74 (16)	4 (57)	81 (18)	4 (44)	62 (24)	14 (47)
	Yes	93 (9)	3 (50)	87 (16)	5 (63)	88 (4)	3 (43)	80 (17)	5 (56)	86 (13)	16 (53)
**Display mode**											
	Light	71 (37)	2 (33)	87 (19)	3 (38)	89 (3)	4 (57)	85 (15)	7 (78)	85 (16)	16 (53)
	Dark	70 (25)	4 (67)	56 (33)	5 (63)	68 (15)	3 (43)	64 (9)	2 (22)	64 (24)	14 (47)

[a]Not applicable.

Table S3. Average scores for each item on the System Usability Scale (SUS) for the different user interfaces (UI).

**SUS item number**	**UI 1**, mean (SD)	**UI 2,** mean (SD)	**UI 3,** mean (SD)	**UI 4,** mean (SD)
1	2.7 (1.2)	3.0 (1.6)	3.1 (1.1)	3.1 (1.5)
2	1.7 (1.2)	2.0 (1.5)	1.7 (1.5)	1.6 (0.7)
3	4.2 (1.0)	3.9 (1.5)	4.4 (1.5)	4.4 (0.9)
4	2.3 (2.1)	3.0 (1.8)	1.7 (1.5)	2.0 (1.6)
5	4.3 (0.8)	4.1 (1.1)	4.7 (0.5)	3.8 (1.4)
6	1.7 (1.0)	2.1 (1.6)	1.4 (0.8)	1.6 (0.9)
7	4.2 (1.2)	4.4 (1.4)	4.9 (0.4)	4.0 (1.4)
8	2.0 (1.3)	2.3 (1.8)	2.1 (1.7)	1.3 (0.7)
9	3.3 (2.0)	3.8 (1.6)	4.0 (1.5)	4.6 (0.9)
10	2.8 (1.8)	2.9 (2.0)	2.1 (1.6)	1.4 (0.7)

Figure S1. Boxplots depicting the distribution of System Usability Scale (SUS) scores grouped by the interface and mobile app use.

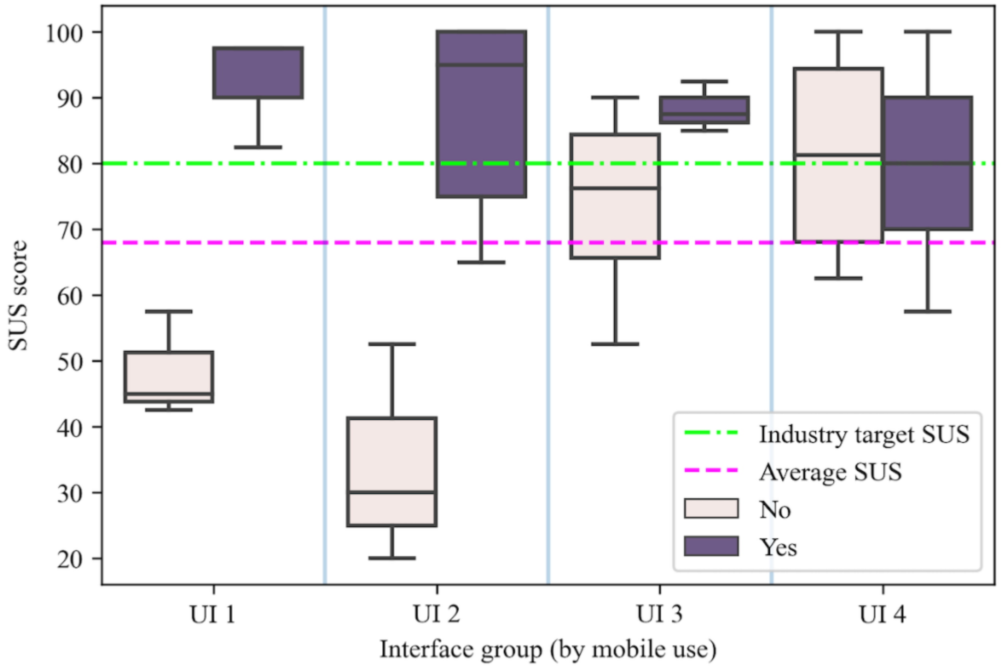

Figure S2. Boxplots depicting the distribution of System Usability Scale (SUS) scores grouped by the interface and display mode.

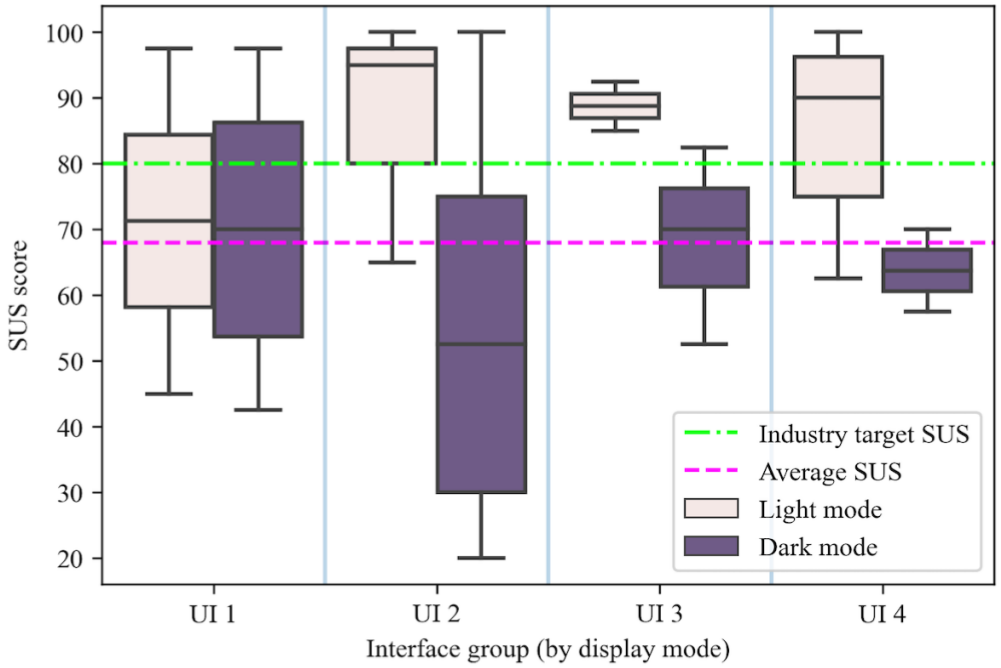

Supplement: Multimedia Appendix 1 [file formative_v8i1e57801_app1.pdf]
